# ‘Leaving no one behind’: reflections on the design of community-based HIV prevention for migrants in Johannesburg’s inner-city hostels and informal settlements

**DOI:** 10.1186/s12889-017-4351-3

**Published:** 2017-05-20

**Authors:** Fiona Scorgie, Jo Vearey, Monique Oliff, Jonathan Stadler, Emilie Venables, Matthew F. Chersich, Sinead Delany-Moretlwe

**Affiliations:** 1 Wits Reproductive Health and HIV Institute (WRHI), Faculty of Health Sciences, University of the Witwatersrand, Johannesburg, South Africa; 2 African Centre for Migration & Society, University of the Witwatersrand, Johannesburg, South Africa; 3 Wellsense International, Kilifi, Kenya; 4 Division of Social and Behavioural Sciences, School of Public Health and Family Medicine, University of Cape Town, Cape Town, South Africa

**Keywords:** Urban, HIV, South Africa, Intervention design, Migrants, Informal settlements, Gender

## Abstract

**Background:**

Unmanaged urban growth in southern and eastern Africa has led to a growth of informal housing in cities, which are home to poor, marginalised populations, and associated with the highest HIV prevalence in urban areas. This article describes and reflects on the authors’ experiences in designing and implementing an HIV intervention originally intended for migrant men living in single-sex hostels of inner-city Johannesburg. It shows how formative research findings were incorporated into project design, substantially shifting the scope of the original project.

**Methods:**

Formative research activities were undertaken to better understand the demand- and supply-side barriers to delivering HIV prevention activities within this community. These included community mapping, a baseline survey (*n* = 1458) and client-simulation exercise in local public sector clinics. The intervention was designed and implemented in the study setting over a period of 18 months. Implementation was assessed by way of a process evaluation of selected project components.

**Results:**

The project scope expanded to include women living in adjacent informal settlements. Concurrent sexual partnerships between these women and male hostel residents were common, and HIV prevalence was higher among women (56%) than men (24%). Overwhelmingly, hostel residents were internal migrants from another province, and most felt ‘alienated’ from the rest of the city. While men prioritised the need for jobs, women were more concerned about water, sanitation, housing and poverty alleviation. Most women (70%) regarded their community as unsafe (cf. 47% of men). In the final intervention, project objectives were modified and HIV prevention activities were embedded within a broader health and development focus. ‘Community health clubs’ were established to build residents’ capacity to promote health and longer term well-being, and to initiate and sustain change within their communities.

**Conclusions:**

To improve efforts to address HIV in urban informal settings, intervention designers must acknowledge and engage with the priorities set by the marginalised communities that live here, which may well encompass more pressing issues associated with daily survival.

## Background

At a global level HIV is increasingly concentrated in cities [1–3]. In sub-Saharan Africa – and in southern Africa in particular – rapid urbanisation is intersecting with generalised HIV epidemics, resulting in remarkably high prevalence and incidence in urban areas [2, 3], and even more so in areas of urban informal housing [4, 5]. Recognition of the urban face of HIV is part of the recent UNAIDS initiative to “fast track” HIV interventions in cities, which sets ambitious targets to support “ending the AIDS epidemic by 2030”. Critically, the UNAIDS initiative emphasises that these targets will only be reached by strategically tackling the income inequality and enormous health disparities that are so common in many of the world’s largest cities. As the UNAIDS Cities Report puts it, this means devising interventions that will “leave no one behind”, instead seeking deliberately to reach “the millions of people who have fallen through the cracks of social, political and economic life” in cities [6].

Within South Africa – which has an estimated 460,000 new HIV infections per year [7] – just over half (50.5%) of all people living with HIV in 2009 were estimated to reside in four cities alone [8]. In 2012, while 18.8% of the country’s reproductive population (15–49 years) was living with HIV, a prevalence of 29.9% was recorded in urban informal areas [7]. This was higher than any other locality type, and double that of urban formal areas (14.7%) – a reminder that cities should not be thought of as homogenous spaces. They are complex, encompass vastly different health and demographic profiles within their boundaries, and are often undergoing rapid change. Even within the category of “urban informal” areas, there is much diversity. In the city of Johannesburg, almost one quarter of the population reside in informal housing, which takes the form of unmanaged municipal hostels; informal shack settlements; illegally occupied, abandoned warehouses and factories; and sub-divided flats controlled by slum landlords [9]. Sometimes described as “hidden spaces” within the city [9], residents in these informal housing arrangements are often marginalised and either invisible to city authorities or deliberately evading intervention. Many are newcomers to the city, migrants in search of work. These “hidden spaces” are also characterised by high unemployment, poverty and overcrowding, alongside very high rates of HIV and TB co-infection [10]. Against this backdrop, the design of urban health programmes is distinctly challenging, particularly where there is intense migration and a highly transient population. Owing to a process of oscillating, or circular, migration, cities such as Johannesburg experience the continuous movement of populations between cities, smaller towns and rural areas [11].

The relationship between migration and infectious disease in southern Africa has garnered intense interest among HIV researchers. While early studies sought to disentangle the routes of HIV spread between city and countryside [12, 13], more recent work has turned to focus on the “social and sexual disruption” wrought by migration and mobility [14]. Such disruption is plainly evident in the social landscape of single-sex hostels in South Africa – in particular, in the epidemiological consequences of large numbers of urban-based male migrants living away from their rural families and wives for long periods of the year [15–17]. Historically, casual sexual relationships with sex workers or more permanent relationships with ‘mistresses’ (*bonyatsi*) in the cities were common [15], as were same-sex relationships within hostels [17]. Data on HIV prevalence amongst urban hostel dwellers in Johannesburg is lacking, but prevalence studies in mining communities such as Carletonville point to extremely high rates of infection among women who live near single-sex hostels [18].

How should responses to HIV be crafted within these internally diverse, highly mobile, informal urban communities, where social conditions are so marked by poverty, gender asymmetries and instability? Our experience suggests that HIV responses must go beyond national, provincial or even city-wide responses, and actively engage with the unique communities living and working within specific urban localities, such as inner-city hostels. This follows a recognition that “HIV prevention programmes must be differentiated and locally-adapted to the relevant epidemiological, economic, social and cultural contexts in which they are implemented” [17, 19]. Furthermore, interventions are more likely to be successful if they are built on genuinely participatory approaches and an understanding of local dynamics of HIV risk and vulnerability.

In this article, we consider the challenge of responding to HIV in the inner-city hostels and surrounding informal settlements of south-eastern Johannesburg, and the role played by community engagement in programme design. We reflect on the process of conducting a formative inquiry (encompassing community mapping, a baseline survey and client-simulation exercise), the results of which were used as a basis for designing and implementing an intervention together with residents in the study setting. Known as *Mpilonhle-Mpilonde* (“Quality life-Long life” in isiZulu), the intervention was led by the Wits Reproductive Health and HIV Institute (formerly RHRU) in 2005, in response to an invitation from the City of Johannesburg, which had expressed concerns about apparent high rates of HIV infection among migrant men in local single-sex hostels. Historically, very few interventions had been delivered to this marginalised population, and the hostels themselves had for long been considered ‘no-go’ areas. The article focus is heavily influenced by our perspectives as researchers and “community outsiders” [20], drawing on our accumulated experience, reflections and discussions over the life of the project.

## Methods

### Study setting

A relatively young city, Johannesburg is now the economic hub of sub-Saharan Africa, with an estimated 4.4 million residents [21]. Since the end of Apartheid, migration patterns have shifted as national borders have opened up and restrictions removed on the movement of South African nationals into and within urban spaces [22, 23]. Roughly three quarters of the city’s population are black African, and English and isiZulu are the two most widely spoken languages [21]. Johannesburg is now an increasingly cosmopolitan city: in 2008, around 14% of its population were estimated to be foreign-born, mainly Zimbabwean, Mozambican and Nigerian [24]. The inner-city, in particular, is home to a heterogeneous population of internal and cross-border migrants, as well as refugees and asylum seekers from across the African continent and beyond [25, 26]. Single-sex hostels in the inner-city provide an entry point to urban life, and are an important source of cheap housing for men (and, more recently, women and children) who have moved to Johannesburg to seek work [27]. Originally created by mining companies to accommodate men from rural areas at minimal cost, hostels were part of an Apartheid strategy to limit the urbanisation of black people from rural *Bantustans*. They have been described as “institutions of incarceration” [28], for their barrack-style environments that were – and remain – typically overcrowded and lacking in privacy [27].

Our study focused on hostels in the Benrose area, in ‘Region F’, south-eastern Johannesburg. These hostels were initially located on the outskirts of the city, but increased urban growth has made them much more centrally located [29]. Following mine closures in the early 1990s, the departure of former employers and the subsequent decay of management structures, the hostels are now occupied by unemployed migrants rather than miners [9]. Over time they have become increasingly unstable, unsafe and unsanitary, characterised by high levels of crime, violence and a lack of functioning amenities. Upgrading programmes devised by the Provincial government, including the transformation of hostels into family units, have had mixed success [30].

### Formative research

Prior to developing the intervention, formative research activities were undertaken to better understand the demand- and supply-side barriers to delivering HIV prevention activities within this community. This included community mapping, a baseline survey, client-simulation exercise in local public sector clinics, and interviews with traditional healers operating near the hostels.

- *Community Mapping and Size Estimation*

Community mapping was carried out in 2003–4 to understand the layout of the study setting and the types of settlements occupied by residents. This was supplemented by participant observation by a trained fieldworker who spent several nights in one of the hostels. Together, these activities provided valuable information on the physical structure and layout of each hostel, as well as the gender dynamics and nature of everyday life within the hostels. It also brought to light the scale of informal settlements located on the periphery of hostel borders, and the complex networks and relationships forged between hostel residents and their neighbours in these settlements.

b. *Simulated Client Visits*

Supply-side issues were elucidated through simulated client visits in 2004 to public sector services located in the vicinity of the hostels. Staff at selected health facilities were informed in advance that simulated clients would be visiting them over the course of the subsequent three months, presenting with the following scenarios: (1) symptoms of an STI; (2) a request for voluntary counselling and testing of HIV (VCT), and (3) enquiries about antiretroviral therapy (ART) for a sick relative. Immediately after their clinic visits, these clients were interviewed in isiZulu by a trained fieldworker about the encounter.

c. *Traditional Healer Interviews*

Traditional healers previously identified during community mapping activities were included in the assessment of local health services if they were recognised by community members as legitimate traditional health practitioners, provided services to residents of the hostels and informal settlements, and had experience with providing care for STIs and/or HIV-related complaints. Semi-structured, in-depth interviews with these healers were undertaken in isiZulu by a trained field worker, and were audio-recorded, translated and transcribed.

d. *Baseline Community and Biological Surveys*

A community-based behavioural survey was undertaken in 2004 with male hostel residents and women living in adjoining informal settlements to collect key baseline data on populations that the intervention would eventually target. Participants aged 18 years and over, and resident in the area for a minimum of 3 months, were randomly selected and invited to complete a socio-demographic questionnaire. Roughly 10% of the overall population of hostels and surrounding settlements were recruited and interviewed (1458 men and 1008 women). Participants were also asked to provide a saliva sample for a linked biological survey assessing Sexually Transmitted Infections (STI) and HIV prevalence (*n* = 2424). For HIV, samples were transported to the laboratory on the same day for testing, which was done using the Vironostika® HIV Uni-Form II Ag/Ab (bioMerieux, Durham, NC, USA).

### Designing the intervention

Our initial intention was to design and implement a peer-education intervention to improve knowledge of STIs and HIV, and increase safer sexual practices among male hostel residents. As results from the formative research made clearer the demographic and health status of the communities in this setting, we began to ask what priorities community members would themselves identify as the basis for an intervention, if given the chance to articulate them. A brief participatory exercise was undertaken to elicit and capture these priorities. Community members from both the hostels and surrounding shack settlements were recruited with the assistance of a community NGO partner working in the hostels, to participate in a pair-wise ranking exercise [31]. In a series of six sessions, participants were placed in groups and asked to record problems faced by their community on blank cards, one problem per card. The list of problems generated by this activity was then read out by a representative from each group. All participants present proceeded to group these problems under ten main categories. Each card was placed next to an appropriate symbol for each category, and comparisons between the categories were made. Participants then generated a final priority list by ranking each of the ten categories according to the number of needs recorded, and by expanding, clarifying or consolidating some of the categories. Results from this exercise were fed directly into the process of intervention design, alongside findings from the formative research phase.

### Process evaluation

Once implementation of the intervention was complete, a process evaluation was carried out to assess fidelity to project planning, and to identify major strengths and weaknesses. This involved an evaluation of actual outputs against a matrix of planned activities and key project objectives for selected components of the project. The ‘Quality Living Clubs’ (QLC) – the only component described here in any detail – were evaluated using a variety of methods, including analysis of attendance registers; use of an attendance tracking tool; assessment of module content, and exit interviews with QLC facilitators and 123 of the 148 ‘graduates’ of the QLCs (those who completed at least one module, i.e. two out of three learning sessions and one out of three reflect circles). Interviews were recorded and transcribed before being entered into Epi-Info, coded, and imported into an Excel spreadsheet for analysis of basic statistics. Qualitative portions of the interview data were reviewed by the research team and thematically analysed to triangulate quantitative findings, particularly on components more difficult to evaluate, namely, fidelity and implementation. Illustrative quotes were also identified from the interview data to highlight key findings.

## Results

### Demand-side factors influencing healthcare needs

**Fig. 1:**
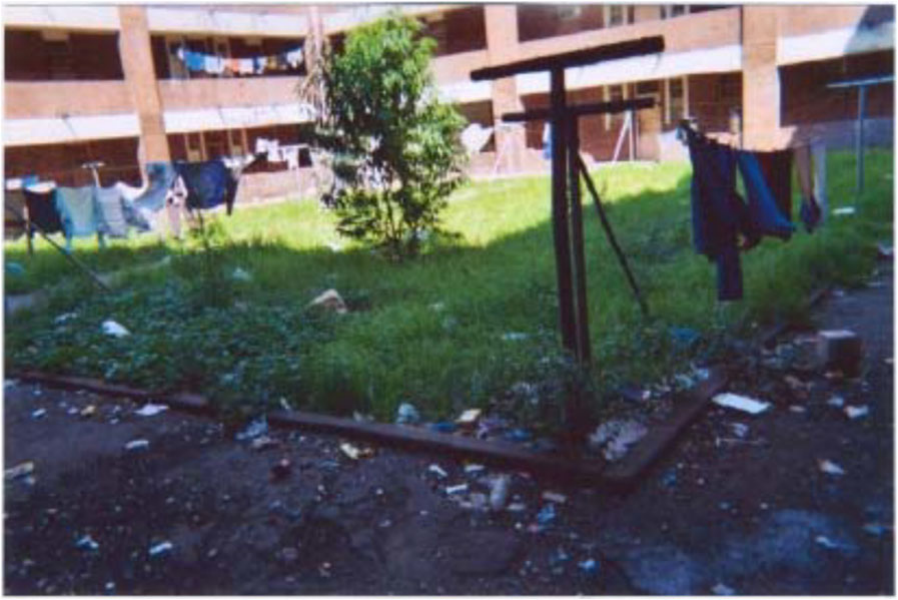
Denver Hostel, Benrose, Johannesburg

Mapping activities confirmed the location and size of six hostels in the intervention area (at the time of the study, four were administered by the municipality and two privately), and identified specific localities within this area associated with high levels of substance abuse. Importantly, the mapping led to a revision of population estimates from an initial figure of 7000 residents to around 30,000. At the time of the intervention, Denver, Jeppe and George Goch Hostels each had between 3200 and 3700 registered residents, but were estimated to house as much as three times as many men (see Fig. 1). Demand for housing adjacent to the hostels was high: abandoned buildings and warehouses located nearby had been taken over by slumlords and subdivided for letting, while people were living in an abandoned sports stadium in the area and in shacks built on former factory plots. These shacks and other informal forms of housing had emerged partly as a result of hostel traditions enforced by the gatekeepers – local *indunas* (IsiZulu: ‘headmen’) – which did not allow women and children within the strongly male-dominated environments of the hostels. These prohibitions were strictly enforced and, according to local informants, women could be raped or assaulted if they were found inside the hostels. Consensual sexual activity with women – i.e. sex within the context of a relationship, or sex with a sex worker – was also reportedly prohibited within the hostels, leading many male hostel residents to construct informal housing nearby for their wives and partners. Our survey showed that around two thirds of men had family members either living in the hostel with them or in neighbouring informal settlements and that despite several years of residence in Johannesburg, both populations still overwhelmingly regarded the city as a temporary location, and saw their ‘home’ as being outside the province. Overwhelmingly, most of the men (94%) were from KwaZulu-Natal province, and described themselves as being of Zulu ethnicity. Half reported travelling to their rural homes at least once a year. Around three quarters of women (76%) were also internal migrants from KwaZulu-Natal (76%).

**Fig. 2:**
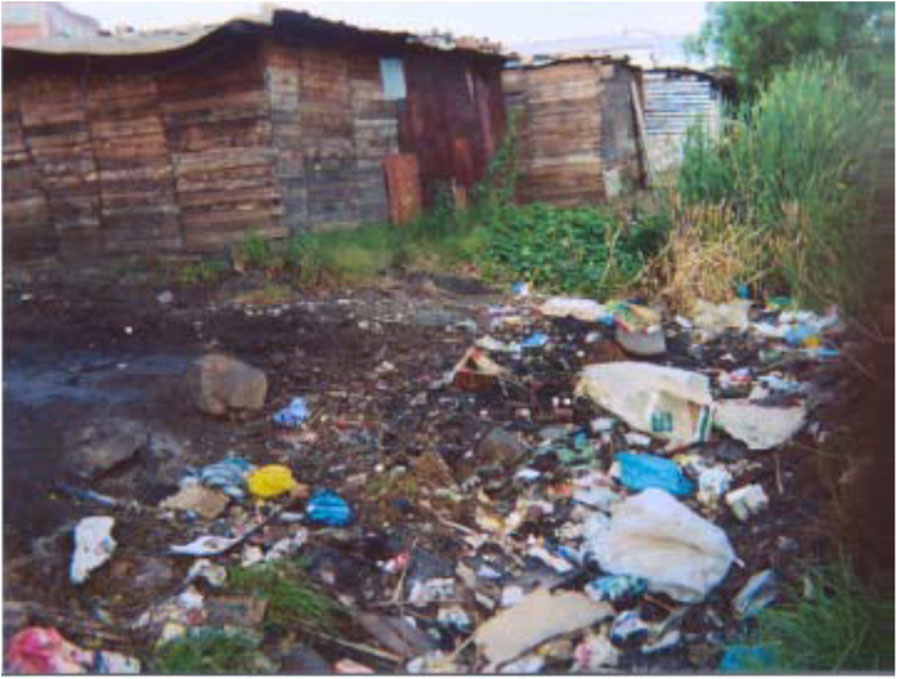
Shacks in Mangolongolo Settlement, Benrose, Johannesburg

Hostel life was largely defined by overcrowded living conditions: 70% of the men surveyed shared a room with at least five other men, and as much as 44% shared with ten or more. Water and sanitation facilities were often lacking, with toilets reportedly unusable at least once in a month. Informal settlements surrounding the hostels were also described as overcrowded, and lacking in adequate sanitation and electricity (see Fig. 2). The latter problem in turn created a demand for alternative cooking fuels, such as paraffin, which posed a potential fire hazard. On average women had not lived in the informal settlements as long as men had been in the hostels: only a third of women had lived there for more than 4 years. Around half of the men (51%) had at least one child, but both men and women reported having to provide for a number of financial dependants on what was, for most, a meagre and erratic income. Some small businesses, such as car maintenance and shoe repairs, had sprung up in these informal settlements, alongside *spaza* shops (small informal grocery shops), *shebeens* (taverns), restaurants and butcheries. Seventy-six per cent of women interviewed and 56% of men stated that they were not working.

Concurrent sexual partnerships were common: at least 47% of men claimed to have more than one sexual partner at the time of the survey, and a notable portion of these men said they had exchanged money (5%) or gifts (10%) for sex. Only 2% of women from the informal settlements reported having been paid for their most recent episode of sex and none identified themselves as “sex workers”. Eighty nine percent of women had boyfriends, who were generally two or more years older than themselves. As measured by biological samples taken during the survey, more than twice as many women (56%) were found to be living with HIV than men (24%). These figures were considerably higher than national HIV prevalence estimates for 2004, the year of the survey (16.6% of men and 20.2% of women aged 15–49) [32], and, indeed, higher than more recent surveillance data from 2012 (14.5% and 23.2% respectively) [7].

Consuming alcohol and marijuana, playing cards and gambling, illicit activities and crime were described as part of the fabric of life in the hostels, with “fighting” and “shooting” reportedly commonplace. The gender dynamics of local interactions and women’s comparatively greater vulnerability to violence were clearly evident: a third to a half of all men considered their community to be unsafe (47%), compared to 70% of women. Around a quarter of women reported ever being beaten by their partner or husband, 11% had ever been threatened into having sex, and 8% had ever been raped. In addition, 6% of men acknowledged that they had ever used physical force against a woman to have sex. Despite these perceptions of a lack of personal safety, residents expressed a strong sense of belonging and community cohesiveness both within the hostels and informal settlements. They described feeling “alienated” from the broader city of Johannesburg, and men said they felt closer to other hostel residents – most of whom were also migrants from the same province – than to people in other parts of the city.

In terms of accessing healthcare, public-sector clinics, health centres and hospitals were most commonly consulted by women and men alike, although men were twice as likely to seek private healthcare as women (14% vs. 7%). Women generally had greater familiarity with available services for STI treatment and HIV testing; four times as many women reported having had an HIV test, compared to men (41% vs. 10%). More than half of women used no contraception, and consistent condom use was also low, with less than a quarter of men and women reporting use of a condom at last sex. Sexual decision-making was perceived by more than three-quarters of women to be the prerogative of their male partners.

### Supply-side barriers to health care

During the assessment of available health facilities, ten government health units were identified within a 2 kilometre radius of the hostels in the study area. In addition, three pharmacists, nine private general practitioners and ten traditional healers were practising in the area. A total of 44 simulated client visits were completed by 12 trained hostel and informal settlement residents (six male, six female) who visited nine public sector health facilities. These visits showed that the greatest challenge to the provision of quality health services in the inner-city of Johannesburg was human resource capacity, in which a lack of qualified staff in clinics led to prolonged waiting times. Attitudes of health care providers to clients were found to vary with the reason for the consultation. Clients enquiring about HIV testing or ART received better care than those presenting with the STI scenario, suggesting that healthcare providers may have looked more favourably upon those proactively seeking health information, and that patients with STI symptoms were likely to be stigmatized. There was a general failure to follow national guidelines on STIs, including the physical examination of patients reporting STI symptoms. Further problems included inappropriate questioning of clients by untrained staff at clinic reception areas and the absence of particular services on some days of the week.

Interviews with traditional healers confirmed that the majority of clients visiting these healers came from the neighbouring hostels and surrounding informal settlements. The healers reported treating male clients primarily for complaints of impotence, backache, “*doropo*”^1^ (gonorrhoea), swollen feet and “*idliso*” (bewitchment, or magical poisoning). In most cases, patients who were believed to be living with HIV were allegedly referred to mainstream public sector clinics for care.

### Weighing up formative results: implications for intervention design

Prior to the collection of formative data, our intention had been to devise an intervention to improve knowledge and awareness of STIs and HIV, and increase safer sexual practices (e.g. partner reduction, increased condom use, HIV testing and seeking symptomatic STI treatment) among men in urban hostel settings. In addition, it was envisaged that the programme would strive to increase residents’ utilisation of primary healthcare (PHC) services for Sexual and Reproductive Health (SRH) needs. Collectively, however, the formative research findings necessitated a radical revision of these objectives.

The mapping exercise, for example, led to an important adjustment of the study parameters. It identified a large population of mostly migrant women living in informal settlements surrounding the hostels, and largely invisible to city authorities. This meant that the intervention could no longer engage male hostel residents alone. Survey findings indicated that local gender dynamics and women’s precarious living arrangements rendered them particularly vulnerable to violence, HIV infection and poor health – suggesting that the intervention needed to approach HIV as part of this nexus of vulnerability and recognise its spatial dimensions. The client-simulation exercise revealed that both women and men experienced poor quality SRH services, thanks largely to inadequacies in staffing and training of health-care workers. There was apparently low demand for these services, as shown by low uptake of contraception among women and low HIV testing rates by all.

**Fig. 3:**
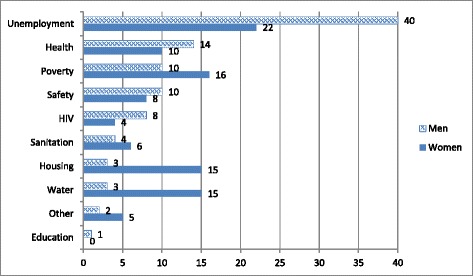
Community-led prioritisation of needs generated through a “pair-wise ranking” [30] approach. “Other” includes: alcohol, shack fires, no toilets or dirty toilets, dirtiness, intermittent or absent electricity, tribalism, frequent death in the community, lack of understanding and information

While we anticipated that the built environment and social context of the hostel setting would shape intervention design, what was unexpected was the extent to which active involvement of local communities would impact on project scope and activities. Through the participatory pair-wise ranking [31] exercise undertaken with community members at the close of our formative research phase, we became aware of the contrasting priorities of men and women living in this setting – and the relatively low priority afforded to HIV (see Fig. 3).

These divergent priorities were driven to some extent by their different living conditions. For men in the hostels, water, sanitation and housing were not as much of a concern as they were for informal settlement residents, and where men prioritised the need for jobs, women identified poverty alleviation more strongly as a priority. To some extent, this may reflect local gender roles, in which men are traditionally the (employed) providers, while women are expected to care for the home and family – roles that are directly impacted on by inadequacies in housing, sanitation and access to water. Women also identified a broad range of everyday issues (included in the “other” category) as concerning, including alcohol abuse, absent or unreliable electricity services and shack fires. In spite of the relatively high HIV prevalence in this community (particularly amongst women), both women and men identified HIV as less of a priority than general health concerns. Instead, most wanted action to be taken on what they saw as more pressing issues associated with daily survival: principally, employment and financial stability.

Incorporating this local input meant that – to be relevant – the programme had to shift from its core concern with HIV to a broader focus on community development and health. During project planning, the study team had thought to use peer-education to promote safer sex practices, but these community-driven insights highlighted the inappropriateness of such an activity in this setting and context. The objectives of the intervention were therefore re-conceptualised to focus more broadly on increasing livelihood security and wellbeing, rather than on promoting safer sex practices and improving SRH services alone.

### Intervention framework

**Fig. 4:**
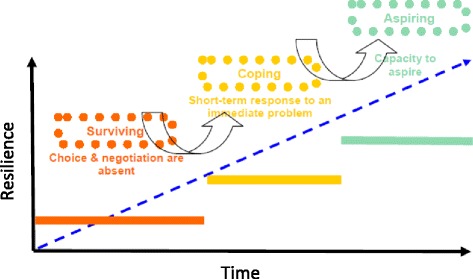
Schematic representation of the Sustainable Livelihoods Approach continuum

Intervention design was partly influenced by the concept of the poor collectively acting to break down structural inequalities and build a ‘capacity to aspire’, as formulated by Appadurai [33], together with Freire’s notion of learning through the creation of a ‘critical consciousness’ [34]. In practical terms, we also drew heavily on a Sustainable Livelihoods Approach (SLA), which primarily aims to expand the level of control that participants have over their lives. The SLA presupposes that individuals occupy a position on a livelihood continuum ranging from surviving (where choice and negotiation are absent) through coping (where short-term solutions to newly identified challenges are possible) to aspiring (which involves planning for the future) [35, 36]. As individuals move along this continuum from surviving to aspiring – although in theory they could also move in the opposite direction, depending on circumstances – they expand their ‘capacity to aspire’ [33]. Accordingly, the level of control over their own lives increases, which in turn builds assets and enables a greater ability to cope with social challenges (see Fig. 4). It was hypothesised that participation in activities to promote health and long term well-being would enhance community members’ resilience to the shocks and stresses associated with migration and urban living, and ultimately enable them to actively engage with HIV and other SRH interventions. In other words, the route towards promoting and entrenching safer sex practices was neither as direct nor as explicit as initially envisaged.

### Mpilonhle-Mpilonde: driving change through community-based health clubs

Ultimately, the intervention comprised three related components and was implemented in 2005–6. These focused on multi-sector, health sector and community sector activities respectively: (1) developing a coordinated response to the community’s diverse priorities through strengthening working relationships between the City of Johannesburg, the Gauteng Provincial government and local NGOs; (2) training healthcare workers to deliver quality, relevant SRH services – in particular, STI treatment; and (3) formation of community-based ‘health clubs’ (known as Quality Life Clubs, or ‘QLCs’) to serve as a central vehicle for driving change in the intervention communities. Only the third component is detailed here, as developing these health clubs drew most heavily on participatory approaches, and embodied most clearly the shift in project focus.

Based on a fusion of several innovative methodologies used elsewhere, including “study circles” [37], community health clubs [38, 39], and the “Reflect” methodology [40], the QLCs developed within *Mpilonhle-Mpilonde* explicitly acknowledged the local contexts of poverty, social marginalisation and the particular vulnerability experienced by urban migrants. Similar clubs have been implemented in rural settings in both Zimbabwe [38] and Sierra Leone [38], as a long-term strategy to enhance people’s control over the determinants of health. In our intervention setting, QLCs were established as voluntary, community-led structures to encourage change through a process of participatory learning and reflecting [41]. In a series of carefully structured “learning sessions” facilitated by a trained, expert facilitator from partner organisations (Idasa, Soul City and The Greenhouse Project), community members identified challenging and problematic aspects of their environment, and collectively formulated responses to bring about change.

Ideally, QLCs would meet weekly over a six-month period for group learning sessions, and then gather later in the week in smaller “reflect circles” [37, 40] to discuss and reflect on what they had learnt that week. To facilitate positive group dynamics, discussions of community needs began with practical, non-sensitive topics, such as environment and sanitation, or food and nutrition, and only moved on to more sensitive topics – such as gender roles, violence or HIV – when club members had acquired sufficient confidence and group management skills to actively engage with these more complex issues. While some reflect circles focused largely on individual capacity-building, like developing *curriculum vitae* writing skills, others were devoted to broader community-focused activities, such as neighbourhood clean-up campaigns, community gardening, support for raising issues with local authorities, and community mobilisation for HIV ‘Know Your Status’ campaigns. Yet other reflect circles made income-generating activities the central focus. These included building skills to make household goods and crafts for re-sale, and support for initiating small-scale catering businesses. In a community made up largely of unemployed migrants, such initiatives became especially important ways of fostering a sense of security, positive community identity, and hope for the future.

Between February 2005 and July 2006, three rounds of this intervention were facilitated by research staff and partners, involving 479 community members. By December 2006, a fourth round was implemented by community members who had been mentored by the research team in earlier rounds. Interestingly, modules focusing on HIV had the highest attendance over the course of the three rounds monitored (652 participants attended at least one learning session or reflect circle, indicating that some participants actually attended these modules more than once), closely followed by those discussing gender and violence (553 attendants; some attended more than once). Women formed the majority at both learning sessions and reflect circles regardless of topic, possibly reflecting local gender norms that assume men will take ownership of projects as leaders, while women will make up the bulk of ordinary participants and volunteers in grassroots community development [20]. Gender also featured strongly in the breakdown of those attending sufficient sessions to qualify for ‘graduation’: 83% of graduates were women.

A key motivation for joining the QLCs, cited by most of the QLC graduates interviewed for the process evaluation, was a desire for knowledge acquisition, particularly knowledge on health issues. One fifth of the members interviewed said that the most important thing they had gained was a sense of empowerment, while 86% of graduates had noted a change in their lives as a result of participation in the programme. One young woman reflected that *“I see things differently now, and my behaviour and lifestyle has changed”* (DIS096, aged 25), while a male participant said, *“[A] lot of things has changed, my behaviour, the way I think and talk”* (JO29, aged 37). Testimony from facilitators and participants alike suggested that women in particular had grown in confidence as a result of their participation in the QLCs. One facilitator of the reflect circles at George Goch Hostel described the transformation he had witnessed in a woman attending these circles. Initially, “…*she was very shy, with no sign she can engage. It is amazing how her confidence has increased”.* Other female participants commented, *“In Reflects, I have learnt to speak out my mind”* (DIS021), and *“I am able to do some of the things that I was not able to do before I joined Quality Life Clubs” (JO23, aged 36).*

Finally, an important question posed of the QLCs in the process evaluation related to their sustainability. While six QLCs had been initiated in 2005, and peer-to-peer recruitment campaigns had even sprung up to encourage other residents to join the QLCs, by 2006 these had been consolidated and only three clubs remained operational. Membership decreased between rounds one and two for all of the QLCs, and some clubs ceased to function altogether, mainly owing to participants leaving to pursue employment opportunities or volunteer in government programmes. There were also challenges in securing a regular meeting venue. Countering these apparent signs of non-sustainability, to some extent, was the fact that at least seven graduates were known to have started their own QLCs after the programme ended. In one instance, there was even a ‘ripple effect’ felt beyond the study setting, as one male migrant exported the concept of QLCs back to his hometown in KwaZulu-Natal shortly after completing the *Mpilonhle-Mpilonde* programme. In the intervention site itself, QLC members took over the programme after the third round, and launched their own community-based organization during the graduation ceremony. This organisation, named ‘*Sipho Esihle’* (‘Precious Gift’) was formed to provide networking support for community projects trying to create sustainable, healthy livelihoods. Overall, as community driven structures, the QLCs and the derivations they later spawned, demonstrated a clear potential to create a sense of community and to build demand for health and civic engagement more broadly.

## Discussion

A consideration of the relationship between urban space and health is not new (for example, see [42, 43]), and in South African urban settings, this relationship is receiving increasing attention in public health research [10, 44]. As in other neglected inner-city areas in the global south, the urban settlements targeted by the *Mpilonhle-Mpilonde* intervention are the products of poor management of urbanisation and the inadequate provision of secure housing for new arrivals to the city. They are also marked by the historical legacy of Apartheid housing policies that institutionalised racial, class and gender inequalities. This is an environment where HIV and STI risk is elevated, health service provision is distinctly inadequate, and social marginalisation creates a series of “interlinked deprivations” [10]. These deprivations – experienced by virtually all sectors of the urban poor in South Africa – in turn function to become potential drivers of the HIV epidemic in cities [10, 45].

Our experience of designing and implementing *Mpilonhle-Mpilonde* revealed the extent to which Johannesburg’s inner-city hostels and surrounding settlements continued to be structured along gendered lines, long after the historical, sex-segregated configuration of these institutions had formally ended. Like residential spaces in many other settings, [46, 47] the hostels and adjacent settlements were invested with gendered meanings, divisions and boundaries that impacted profoundly on the nature of everyday life and, consequently, on individual health outcomes. From vulnerabilities to violence, to the varying prioritisation of community needs, it was impossible to ignore how gender and space shaped the lives of women and men living here, and how perceptions of HIV risk were constructed accordingly. Equally, high unemployment, strong ties to their home communities and limited allegiance to the city underpinned residents’ frustration with their lives in Johannesburg. Livelihood insecurity and dependency on others for wellbeing led to a lack of integration into city life and a deepening of the sense of isolation so familiar in migrant communities.

The eventual orientation of the intervention – with its activities embedded in a broader health and development focus – flowed directly from the input of local residents in this unique urban space. Community members set out their priorities and concerns in various participatory fora, thereby demonstrating the urgency of enhancing the capabilities of residents to articulate their needs and shift the conditions of their lives, from whence they can potentially engage with health-promoting activities as more active, empowered participants. The decision to ground the community health clubs (the QLCs) in a Sustainable Livelihoods Approach made the intervention particularly responsive to the gendered dynamics of the setting, as evidenced by women’s testimonies upon emerging from the QLCs with greater confidence to engage actively in community development.

Although the *Mpilonhle-Mpilonde* project was developed almost a decade prior to the launching of the UNAIDS “Fast-Track Cities” initiative, it in fact foreshadowed and encapsulated many of that initiative’s key principles. Most critical among these is the recognition that equipping people to protect themselves against infection and ill-health often requires interventions to be structured in such a way that they explicitly tackle broader social and economic inequity and marginalisation. And in practical terms, according to UNAIDS, efforts to prevent and control HIV will be more effective when they are built on greater social inclusion: “when they are rights-based, and when they involve the active participation of affected communities” [6, 19]. These ideas formed the core of the *Mpilonhle-Mpilonde* project, and its focus on building the capabilities of marginalised communities to take control of their lives and health, and thereby take steps to protect themselves against HIV infection. Closely related to this approach, the use of participatory methods to identify community priorities and needs resulted in residents’ greater involvement and ownership of the project, and ultimately shifted the focus of activities to take account of people’s actual circumstances. These principles may seem obvious upon reflection, but they continue to be surprisingly absent in HIV interventions in practice.

The UNAIDS Cities Initiative also makes much of the particular advantages held by cities, and which – it argues – must be seized if HIV interventions in these settings are to be effective [6]. These advantages include the networking of talent, knowledge and resources in urban centres; the more tolerant political and social culture in cities compared to rural areas; and the fact that in theory, at least, it is easier to reach people with information and services in cities. As the *Mpilonhle-Mpilonde* experience showed, the rapidly growing urban informal housing sectors in Johannesburg’s inner-city have dynamics of their own that may complicate attempts to capitalise on such advantages. Characterised by high unemployment and inequality, and with a somewhat transient population of people new to the city, these areas have come to host pockets of marginalised communities that are all but forgotten by local authorities. Yet maximising the advantages identified by the UNAIDS Cities Initiative presupposes the existence of relatively empowered communities with the requisite social capital for accessing available resources, networks and knowledge. Interventions such as *Mpilonhle-Mpilonde* may well serve to enable highly marginalised, urban migrant communities to tap into broader networks in the city, thereby taking the first steps towards lowering their HIV risk.

### Study limitations

Several limitations in project design and implementation must be noted. Firstly, even with an intense focus on community participation, the project team was not able to secure optimal levels of participation from residents, and there was a steep drop-off in participant numbers over time. Of the 479 participants attending the QLCs, only 148 graduated. Clearly, even with its broadened focus on building capacities and improving wellbeing (rather than on narrowly promoting access to SRH services), the intervention was still not a priority for many residents. Searching for formal employment, for example, demanded much of their time and effort, making active and sustained participation in this intervention a challenge. Secondly, it was not possible to evaluate the full impact of the intervention on the community, partly because the project was never meant to encapsulate a rigorous trial of an intervention, but also because funding constraints made it impossible to implement a full post-intervention evaluation, and only allowed for a limited process evaluation. Thirdly, the data on which this paper draws is now a decade old, a decade during which the HIV intervention landscape has changed substantially. Access to ART via the public health system has improved considerably, and several new prevention methods have been developed, such as oral Pre-Exposure Prophylaxis (PrEP) and voluntary Medical Male Circumcision (MMC). Yet the lessons learned from *Mpilonhle-Mpilonde* are arguably as relevant today as they were in 2005. Gaps in access remain: the urban spaces associated with the project remain mostly unchanged since it was undertaken, and HIV incidence and prevalence continues to be highest within urban informal areas – as reflected in the initial baseline survey. Furthermore, the delay in publication of these important data reflect a number of realities associated with the financing, planning and implementation of public health programmes that are reliant on grant funding. Staff turn-over and adjustments to the priorities of funded projects affect the ability to streamline implementation, monitoring and evaluation and, ultimately, publication.

## Conclusion

In conclusion, whilst the formative research and intervention design described here was carried out in Johannesburg, a city whose complex history and migratory flows make it a distinct urban space, the experience is nonetheless relevant to other urban contexts. Specifically, the lessons learned in our project may inform the design of effective responses to HIV in marginalised inner-city areas, especially where institutionalised urban housing is in place and where daily life is structured by stark gender differences. The study highlights some of the challenges inherent in engaging with the local, developmental context of unemployed migrants seeking to create a livelihood in the inner-city. Residents of such spaces confront a range of challenges and it should be expected that they will prioritise access to employment and housing above HIV and other health conditions. To improve efforts to address HIV in these settings, intervention designers must acknowledge and engage with the priorities set by the marginalised communities that live here, which may well encompass more pressing issues associated with daily survival.

## Abbreviations

ART: Antiretroviral therapy
MMC: Medical male circumcision
NGO: Non-governmental organisation
PHC: Primary health care
PrEP: Pre-exposure prophylaxis
QLC: Quality life club
SLA: Sustainable livelihoods approach
SRH: Sexual and reproductive health
STI: Sexually transmitted infection
VCT: Voluntary counselling and testing

## Footnotes

1 From the Afrikaans word “dorp”, meaning “town”, this term came to be used when migrant men returned to their rural homes with sexually transmitted infections, which in turn became associated with life in the town.

## References

[1] Dyson T (1993). HIV/AIDS and urbanization. Popul. Dev. Rev.

[2] Montgomery MR. Urban poverty and health in developing countries. Popul. Bull. 2009;64(2).

[3] Garcia-Calleja J, Gouws E, Ghys P (2006). National population based HIV prevalence surveys in sub-Saharan Africa: results and implications for HIV and AIDS estimates. Sex Transm Infect.

[4] Shisana O, Rehle T, Simbayi L, Parker W, Zuma K, Bhana A, Connolly C, Jooste S, Pillay V (2005). South African National HIV Prevalence, HIV Incidence, Behaviour and Communication Survey, 2005.

[5] Kyobutungi C, Ziraba A, Ezeh A, Ye Y (2008). The burden of disease profile of residents of Nairobi's slums: Results from a Demographic Surveillance System. Popul Health Metrics.

[6] UNAIDS (2014). The Cities Report.

[7] Shisana O, Rehle T, Simbayi LC, Zuma K, Jooste S, Zungu N, Labadarios D, Onoya D (2014). South African National HIV Prevalence, Incidence and Behaviour Survey, 2012.

[8] van Renterghem H, Jackson H. AIDS and the city: intensifying the response to HIV and AIDS in urban areas in sub-Saharan Africa. Durban: UNAIDS; 2009.

[9] Vearey J (2010). Hidden Spaces and Urban Health: Exploring the Tactics of Rural Migrants Navigating the City of Gold. Urban Forum.

[10] Vearey J, Palmary I, Nunez L, Drime S (2010). Urban health in Johannesburg: the importance of place in understanding intra-urban inequalities in a context of migration and HIV. Health Place.

[11] Lurie M, Cohen R (2003). The epidemiology of migration and AIDS in South Africa. Migration and health in Southern Africa.

[12] Lurie MN, Williams BG, Zuma K, Mkaya-Mwamburi D, Garnett GP, Sweat MD, Gittelsohn J, Karim SS (2003). Who infects whom? HIV-1 concordance and discordance among migrant and non-migrant couples in South Africa. AIDS.

[13] Lurie M, Harrison A, Wilkinson D, Abdool Karim S (1997). Circular migration and sexual networking in rural KwaZulu/Natal: implications for the spread of HIV and other sexually transmitted diseases. Health Transit. Rev.

[14] Crush J, Williams BG, Gouws E, Lurie M (2005). Migration and HIV/AIDS in South Africa. Dev South Afr.

[15] Jochelson K, Mothibeli M, L JP (1991). Human Immunodeficiency Virus and Migrant Labor in South Africa. International Jurnal of Health Services.

[16] Lurie M, Williams BG, Zuma K, Mkaya-Mwamburi D, Garnett GP, Sturm AW, Sweat MD, Gittelsohn J, Abdool Karim SS (2003). The Impact of Migration on HIV-1 Transmission in South Africa : A Study of Migrant and Nonmigrant Men and Their Partners. Sex Transm Dis.

[17] Moodie TD (1988). Migrancy and Male Sexuality on the South African Gold Mines. J South Afr Stud.

[18] Zuma K, Gouws E, Williams BG (2003). Risk factors for HIV infection among women in Carletonville, South Africa: migration, demography and sexually transmitted diseases. Int J STD AIDS.

[19] UNAIDS (2005). Intensifying HIV prevention: UNAIDS policy position paper.

[20] Campbell C, Gibbs A, Maimane S, Nair Y (2008). Hearing community voices: grassroots perceptions of an intervention to support health volunteers in South Africa. SAHARA J.

[21] Statistics by Place: City of Johannesburg [http://www.statssa.gov.za/?page_id=1021&id=city-of-johannesburg-municipality]. Accessed 12 Jan 2017.

[22] Crush J, Balbo M (2005). Johannesburg, South Africa: Breaking with isolation. International Migrants and The City.

[23] Landau L (2006). Protection and dignity in Johannesburg: shortcomings of South Africa’s urban refugee policy. J Refug Stud.

[24] Centre for Development and Enterprise: Immigrants in Johannesburg: Estimating numbers and assessing impacts. In. Johannesburg: 2008.

[25] Beavon K (2004). Johannesburg: The making and shaping of the city.

[26] Landau LB, Gindrey V (2008). Migration and Population Trends in Gauteng Province 1996–2055. Migration Studies Working Paper Series #42.

[27] Minnaar A (1993). Communities in isolation: Perspectives on hostels in South Africa.

[28] Crush J. Scripting the compound: power and space in the South African mining industry. Environ. Plan.I): Society and Space. 1994;12:301–24.

[29] Thurman S (1997). Umzamo: improving hostel dwellers’ accommodation in South Africa. Environ Urban.

[30] Benit-Gbaffou C, Mathoho M (2010). A Case Study of Participation in the City Deep Hostel Redevelopment.

[31] Chambers R (1988). Direct matrix ranking (DMR) in Kenya and West Bengal.

[32] Dorrington RE, Bradshaw D, Johnson L, Budlender D (2004). The Demographic Impact of HIV/AIDS in South Africa: National Indicators for 2004.

[33] Appadurai A, Rao V, Walton M (2004). The Capacity to Aspire: Culture and the Terms of Recognition. Culture and Public Action: A Cross-Disciplinary Dialogue on Development Policy.

[34] Freire P (1973). Education for Critical Consciousness.

[35] Chambers R, Conway G (1992). Sustainable Rural livelihoods: Practical concepts for the 21st Century. IDS discussion paper 296.

[36] Carney D (1999). Approaches to Sustainable Livelihoods for the Rural Poor.

[37] Idasa: From Idea to Action: A Study Circle Workbook on Starting and Running a Project. Cape Town: Idasa; 2004.

[38] Waterkeyn J, Cairncross S (2005). Creating demand for sanitation and hygiene through Community Health Clubs: A cost-effective intervention in two districts in Zimbabwe. Soc Sci Med.

[39] Waterkeyn J. Cost-effective health promotion: Community health clubs. In: 29th WEDC International Conference. Abuja; 2003.

[40] Newman K (2004). Reflect, rights and governance: Insights from Nigeria and South Africa.

[41] Vearey J, Oliff M, Gardner J, Mbatha T, Cebekhulu V, Delany S (2003). Quality of Life – Long Life: questions raised while gaining access to inner-city hostels and informal settlements for research and interventions. Reproductive Health Priorities Conference.

[42] Vlahov D, Freudenberg N, Proietti F, Ompad DC, Quinn A, Nandi V, Galea S (2007). Urban as a Determinant of Health. J. Urban Health.

[43] Freudenberg N, Galea S, Vlahov D (2005). Beyond urban penalty and urban sprawl: back to living conditions as the focus of urban health. J Community Health.

[44] Stadler J, Delany S (2006). The 'healthy brothel': the context of clinical services for sex workers in Hillbrow, South Africa. Cult. Health Sex.

[45] Venables E, Vearey JL, Cordier M, Oliff M, Rees H, Delany-Moretlwe S (2010). “Know your Epidemic, Know Your Response”: The Need to Contextualize HIV Interventions in Urban Informal Johannesburg. International Conference on Urban Health.

[46] Carsten J, Hugh-Jones S (1995). About the House: Lévi-Strauss and beyond.

[47] Bourdieu P, Low SM, Lawrence-Zúñiga D (1973). The Berber House. The Anthropology of Space and Place: locating culture.

